# Supplementary material for: The interactome of intact mitochondria by cross-linking mass spectrometry provides evidence for coexisting respiratory supercomplexes
Source: Mol Cell Proteomics. 2017 Dec 8;17(2):216–32. doi: 10.1074/mcp.RA117.000470 (PMC5795388; doi:10.1074/mcp.RA117.000470)
Supplement: Supplemental Data [file supp_RA117.000470_133922_0_supp_23978_fzffwf.zip › spectra_annotation/mito_DR_spectra_annotation/434-1-7-1-125-1.pdf]

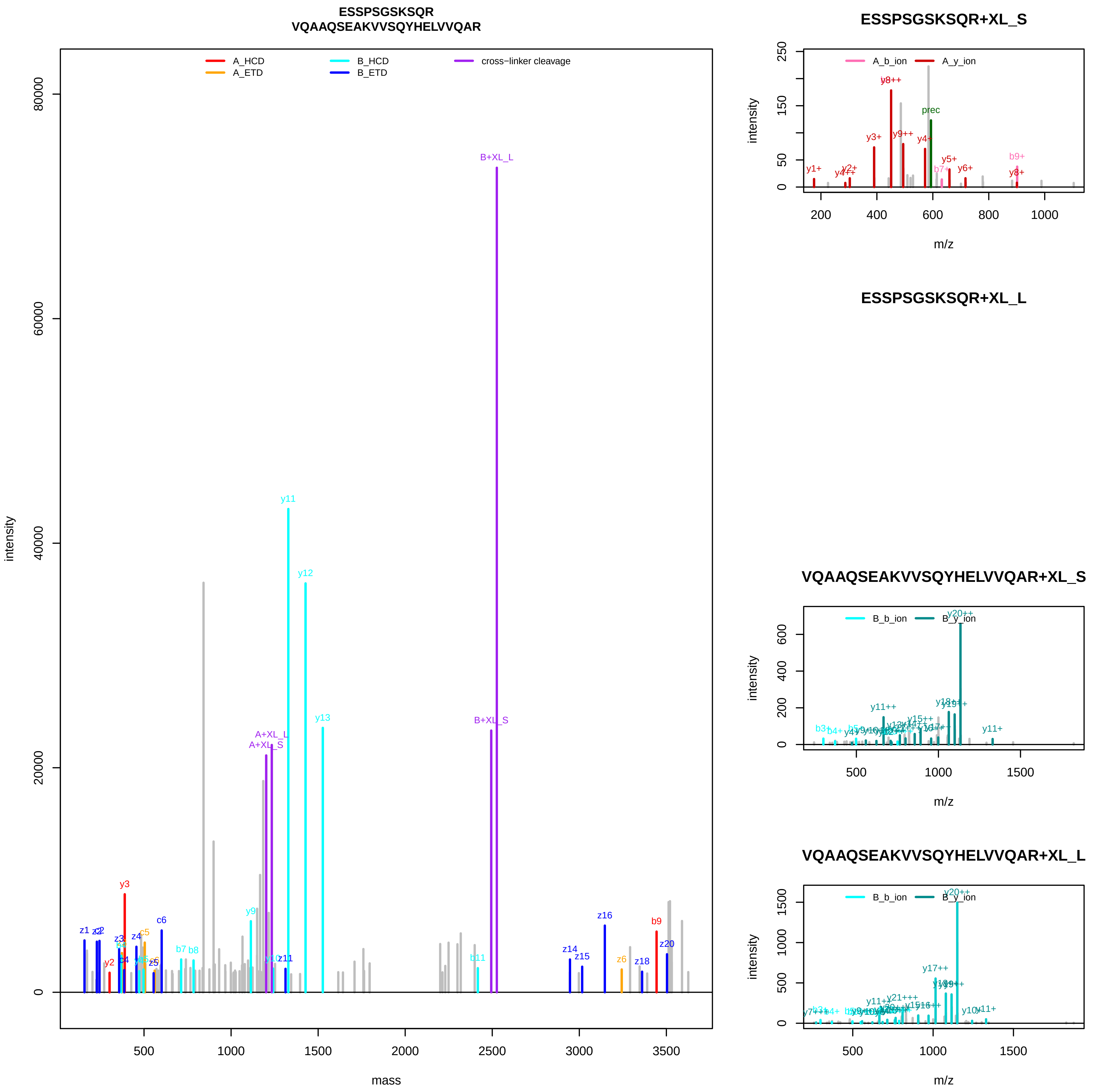
ESSPSGSKSQR
VQAAQSEAKVVSQYHELVVQAR
A_HCD
A_ETD
B_HCD
B_ETD
cross−linker cleavage
intensity
mass
0
20000
40000
60000
80000
500
1000
1500
2000
2500
3000
3500
B+XL_L
B+XL_S
A+XL_L
A+XL_S
y11
y12
y13
y9
y3
y2
b9
b7
b8
b11
c6
c5
z1
z3
z4
z5
z11
z14
z15
z16
z18
z20
z6
ESSPSGSKSQR+XL_S
A_b_ion
A_y_ion
prec
y1+
y2+
y3+
y4+
y5+
y6+
y8+
y8++
y9++
b7+
b9+
m/z
200
400
600
800
1000
0
50
150
250
ESSPSGSKSQR+XL_L
VQAAQSEAKVVSQYHELVVQAR+XL_S
B_b_ion
B_y_ion
y20++
y11++
y11+
y15++
b3+
b4+
0
200
400
600
500
1000
1500
VQAAQSEAKVVSQYHELVVQAR+XL_L
y17++
y21+++
y11+
0
500
1000
1500
